# Ungulate Detection and Species Classification from Camera Trap Images Using RetinaNet and Faster R-CNN

**DOI:** 10.3390/e24030353

**Published:** 2022-02-28

**Authors:** Alekss Vecvanags, Kadir Aktas, Ilja Pavlovs, Egils Avots, Jevgenijs Filipovs, Agris Brauns, Gundega Done, Dainis Jakovels, Gholamreza Anbarjafari

**Affiliations:** 1 Institute for Environmental Solutions, LV-4126 Cēsis, Latvia; alekss.vecvanags@vri.lv (A.V.); jevgenijs.filipovs@vri.lv (J.F.); agris.brauns@vri.lv (A.B.); dainis.jakovels@vri.lv (D.J.); shb@ut.ee (G.A.); 2 iCV Lab, Institute of Technology, University of Tartu, 51009 Tartu, Estonia; kadir.aktas@ut.ee (K.A.); ilja.pavlovs@ut.ee (I.P.); 3 Forest Owners Consulting Center LCC, LV-4101 Cēsis, Latvia; 4 Latvian State Forest Research Institute “Silava”, LV-2169 Salaspils, Latvia; gundega.done@silava.lv; 5 PwC Advisory, 00180 Helsinki, Finland; 6 Faculty of Engineering, Hasan Kalyoncu University, Gaziantep 27410, Turkey

**Keywords:** RetinaNet, Faster R-CNN, animal detection, camera traps, ungulates

## Abstract

Changes in the ungulate population density in the wild has impacts on both the wildlife and human society. In order to control the ungulate population movement, monitoring systems such as camera trap networks have been implemented in a non-invasive setup. However, such systems produce a large number of images as the output, hence making it very resource consuming to manually detect the animals. In this paper, we present a new dataset of wild ungulates which was collected in Latvia. Moreover, we demonstrate two methods, which use RetinaNet and Faster R-CNN as backbones, respectively, to detect the animals in the images. We discuss the optimization of training and impact of data augmentation on the performance. Finally, we show the result of aforementioned tune networks over the real world data collected in Latvia.

## 1. Introduction

The rapid expansion of ungulates has been observed in many European forest regions since the 2000s as the upshot of a complex of factors, including decreased hunting activity and changes in legislation regarding poaching and abandonment of land [1]. Although ungulates, as natural inhabitants of the forest region, usually benefit the forest ecosystem by improving germination conditions for seeds and affecting the forest development, evidence suggests that excessive ungulate density leads to ecosystem disturbance, forestry damage and disease propagation acceleration [2]. The European Wilderness Society reports that annual damage caused by ungulates in the agricultural and forestry sector in EU countries exceeds 100 million euros, suggesting that this problem already causes significant economic losses and should be addressed. Additionally, the overabundance of ungulates increases the danger on the highways and is still an unresolved problem, as there are, on average, 750,000 vehicle collisions with ungulates per year in Europe [3]. As part of the solution to these problems, ungulate population control strategies and monitoring systems should be developed and studied. Monitoring systems should estimate ungulate population distribution, dynamics and ecological change indicators and should manage ungulate overabundance efficiently and systematically.

One of the most common animal monitoring approaches is the camera trap systems, camera traps have been widely used in ecology for wildlife observation and monitoring [4,5,6] due to their non-intrusive nature, ease of use, reliability and cost-effectiveness [7]. A drawback of using camera traps can be the accumulation of large amounts of images or videos that have to be manually sorted and classified [8,9]. Machine learning models have been used to alleviate this task and it has been shown that it can perform as good as or, in some cases, even better than human-made classifications [9,10,11,12,13]. Recently, computer-vision- and deep-learning-based methods have become popular in camera trapping to automatically identify animal species, counts, behaviors and demographic compositions. Norouzzadeh et al. [8] conducted experiments using popular deep neural network architectures and obtained 93.8% classification accuracy on the Snapshot Serengeti dataset in Africa. Carl et al. [14] used a pre-trained FasterRCNN + InceptionResNetV2 network to classify European mammals with a 94% detection accuracy and a 71% species classification accuracy, but did not separate animals from the Deer *Cervidae* family in the model. Another study by Choinski et al. [15] used a YOLOv5 network that also classified Red deer and Roe deer, with F1-scores of 0.86 and 0.58, respectively.

Although deep-learning-based methods have shown promising results for the classification of the ungulates, there is still a large research gap in the topic. Firstly, there is only a limited number of datasets, since taking images of wild animals requires a setup that is difficult to prepare. Secondly, imbalanced datasets are normal to encounter due to the nature of the task and dataset collection; therefore, the impacts of data augmentation on performance need to be studied further. Thirdly, localization of the animal in the image is still a challenging task and performance should be improved.

The lack of data causes a lack of generalization for the developed methods. Consequently, it lowers the performances in real world situations. Thus, we had motivation to collect a new dataset and present our experiments as a baseline for further studies. We collected a dataset that contains images of deer and wild boar in the wild. Our dataset was obtained from 8080 videos recorded over four years. This dataset provides a good resource for the ungulate studies. Moreover, we present a three-module deep neural network architecture to perform localization and classification of the species. We investigated the performance of different backbones on our dataset. We also present the effect of data augmentation on the performance. There are three main contributions in this paper, as follows:

- We compiled new ungulates in the wild dataset that was collected over four years.
- We compared the performance of different backbones in a three-module architecture on the new dataset. Thus, we created a baseline accuracy for animal localization and classification tasks on the new dataset.
- We investigated the effect of data augmentation on the performance.

## 2. Related Work

In parallel with the increasing concern about the extinction of rare animal species and availability of high computational power, animal detection in the wild has become a popular research topic in the last decade. Automated detection methods have been developed to address the issues with the huge amount of data and manual analysis. Deep-learning-based methods hold great importance in detection tasks and perform successfully in various domains [12,16,17,18]. The proven performance of deep learning techniques directed researchers to study deep neural networks (DNNs) in respect to the animal detection task. In recent years, many studies have been conducted to address different challenges related to this task, demonstrating different DNN-based methods.

The most successful results were obtained using CNN-based architectures. Norouzzadeh et al. [8] presented a method which subdivides the classification into two subsequent modules, i.e., detecting an image containing an animal (VGG is selected as the best performing model) and animal classification (ensemble of models). Their method classifies the animals performing on the same level as a crowd-sourced team of volunteers. Later, Christin et al. [14] presented another CNN-based method which utilizes FasterRCNN and InceptionResNet as backbone. After pre-training their network on Open Images Dataset V4 [8], they could achieve 93% accuracy for detecting the highest taxonomy rank animals among the European wild mammal species. Higher accuracy was achieved on custom datasets. The authors of [13] demonstrated a ResNet-18 model that obtained 97.6% top-one accuracy and more than 99.9% top-five accuracy, which are the highest accuracy scores obtained on a custom dataset to date of the publication.

Mohammed et al. [19] approached the problem from a speed perspective and proposed a pipeline to accelerate species identification. They counted on the camera trap images’ harnessing advantages of active deep learning and transfer learning assessing small project data limitation problems. Their experiment with different sample selection strategies for the active learning phase and the k-Center strategy was reported to be the most successful, obtaining 92.2% accuracy.

From a different perspective, Zhang et al. [20] aimed to leverage the temporal information from the camera trap videos and proposed an iterative, embedded graph cut (IEC) method for detecting the regions that potentially contain the animal. Considering regions with intersection over union (IoU) higher or equal to 0.5 overlaps with ground-truth boxes as positive, the proposed system demonstrated an 83.98% average F-score outperforming the Faster-RCNN region proposal by 3.5% and YOLO by ∼8%.

## 3. Methodology

### 3.1. Detection Neural Networks

Inspired from the works mentioned in Section 2, we experimented with two different detection structures in this paper. These are called one-stage detection and two-stage detection or region-proposal detection. As the name suggests, one-stage detection represents a holistic structure, usually in large sequential CNNs (e.g., YOLO, SSD), which generates all predictions by a single run, whereas two-stage detection divides detection tasks into a region-proposal stage and region-classification stage.

In both cases, the general structure of our detector involves image embedding (obtaining low-dimensional image representation), object localization and classification. Object localization is learned by the regression of bounding box coordinates and classification is learned by minimizing classification loss. The detector model architectures could be subdivided into the following three functional modules:

1. **Backbone network**—DNN consisting of convolutional layers which are used for the feature extraction from the input image. Usually, backbone networks which are pre-trained on a natural image dataset, such as ImageNet, are used. Common networks used as the backbone are ResNet50 [21], VGG16 [22], Inception-ResNetV2 [23] and DarkNet-19 [24].
2. **Neck**—DNN module on top of the backbone network. The neck network takes and processes inputs from the different layers of the backbone, harnessing advantages of data pattern distribution over different feature map scales by using FPN (Feature Pyramid Network) [25].
3. **Head**—A feed-forward neural network which performs the classification or regression task. The detector could have multiple heads for performing different classification and regression tasks.

The detector outputs the bounding boxes with the corresponding labels and confidence scores. The confidence score is calculated from the classification head’s last output layer by applying the softmax function, which normalizes the output to the probability distribution [26].

**Figure 1:**
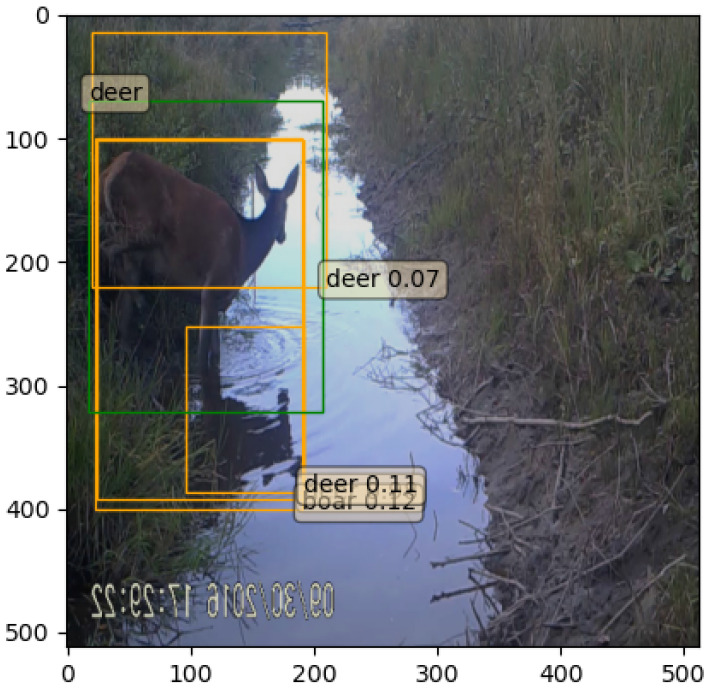
Faster R-CNN predictions before non-maximum suppression. Faster R-CNN produces redundant, overlapping bounding boxes and bounding boxes with low confidence scores. The orange rectangles show the model predictions and the green rectangle shows the ground-truth bounding box.

**Figure 2:**
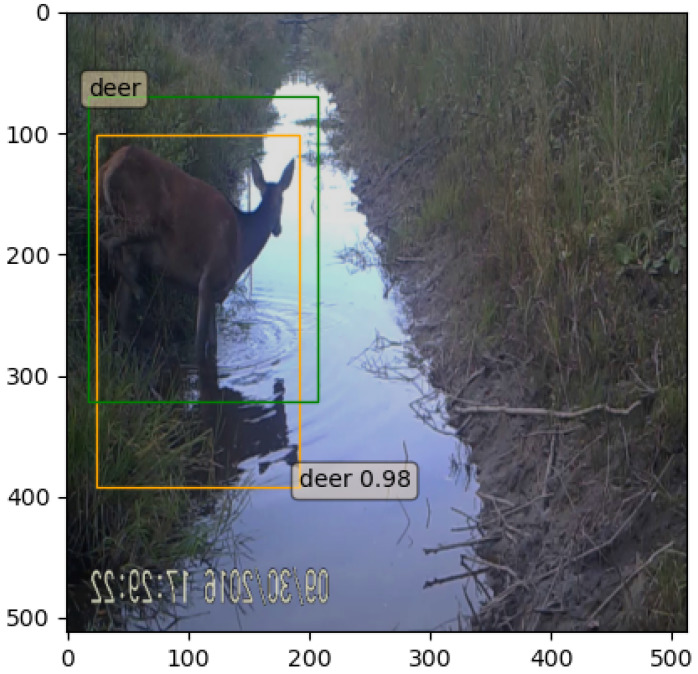
Faster R-CNN predictions after NMS is applied with threshold filtering and different species overlapping criterion. Image reflection is interpreted as the part of the animal.

The detector can generate redundant, overlapping predictions for the same object and produce low-confidence predictions which can be overcome with the non-maximum suppression proposal filtering technique. This method uses the IoU metric to measure how accurately objects are superimposed on each other. The IoU is calculated by dividing the prediction intersection area with their union area, which produces a result between 0 (predictions do not overlap) and 1 (predictions are perfectly superimposed). If the IoU exceeds a certain threshold, the bounding box with a lower confidence score is excluded. Additionally, bounding boxes with small confidence scores could be filtered to exclude misclassified regions. It was observed, in this work, that the detector frequently successfully localized the animal but produced both “boar” and “deer” class bounding boxes around the object. In order to address this issue, a species overlapping criterion was added, which excluded the prediction with the least confidence score in case of multiple overlapping species prediction (see Figure 1 and Figure 2).

### 3.2. One-Stage Detectors

The most popular one-stage detectors are SSD, YOLO and RetinaNet detectors [27,28,29]. YOLO predicts pre-defined anchor boxes for each cell with the associated confidence of containing the object inside the bounding box and offset prediction. It also predicts the probability distribution of classes for each bounding box. Although YOLO produces many outputs, the confidence threshold filters out most of the bounding boxes. The recent updates YOLOv3 YOLOv4 provided significant speed and accuracy improvement and introduced beneficial techniques such as anchor learning, mosaic augmentation and Mish activation, achieving state-of-the-art results of 65.7% mAP@0.5 for the COCO dataset [27]. YOLO is a fast model, which makes it preferable for real-time detection tasks, but it can struggle with small objects and be less accurate than Faster R-CNN.

Another one-stage detector, RetinaNet, stands out for its Focal Loss function, which is used to compensate the foreground–background class imbalance, which was assumed to be the significant problem that produced the one-stage and two-stage detector accuracy gap.

### 3.3. Two-Stage Detectors

The idea behind two-stage detectors is to propose regions where objects are potentially located and then iterate over them and perform classification and bounding box regression (bounding box coordinate offset prediction) by minimizing the corresponding loss functions. Two-stage detector Faster R-CNN precursors (R-CNN and Fast R-CNN) rely on the unsupervised Selective Search algorithm for region proposal. However, the Selective Search algorithm is a fixed algorithm that bottle-necked the Fast R-CNN, limiting the detector’s speed. This limitation was overcome with Faster R-CNN, which introduced the RPN (Region-Proposal Network) for generating regions that provided significant run-time improvement, which allowed Fast R-CNN to be used for real-time detection [30].

In Faster R-CNN model architecture, the feature maps are obtained by running the input image through the convolution layers of the backbone network. Then, region proposals are generated by RPN onto feature maps. Each region is reshaped with an ROI pooling layer and passed to the model head, which performs classification and regression. RPN learns to propose the regions by minimizing the objectness loss and regressing the proposed regions. The whole Faster R-CNN network has four losses, two losses for RPN learning and two losses for detector learning.

## 4. Database: Preparation and Pre-Processing

### 4.1. Dataset

The dataset of interest was collected in Latvia where four wild ungulate species are present—red deer (*Cervus elaphus*), roe deer (*Capreolus capreolus*), elk (*Alces alces*) and wild boar (*Sus scrofa*). Data consisted of 8080 videos in 1280 × 720 resolution and 30 FPS taken over four years. Videos were taken in the daylight and, at night time, using infrared lighting. Data examination revealed that many recordings were falsely triggered (did not contain animals) and the remaining data mostly contained big mammals with a predominance of deer species.

**Figure 3:**
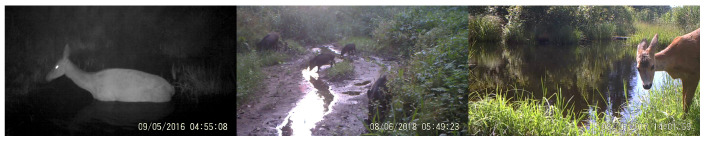
Test dataset samples.

For this study, only wild boar (*Sus scrofa*) and deer (*Cervidae* family) representatives were chosen as the main species of interest. Thus, a smaller subset of the dataset featuring the mentioned species was composed (see Table 1). Labelled images were obtained by sampling annotated videos, resulting in 1128 annotated images featuring both day- and nighttime captures and various object detection hazards, including motion blur, illumination variation, reflections, glare and limited visibility of animals. Samples from the dataset are visualized in Figure 3. Due to the small size of the obtained dataset, we used it as our test dataset in this paper.

### 4.2. Training Data

In order to create our training data, we combined multiple datasets. Since the different datasets contained different levels of taxonomy, we fused the animal species under the *Cervidae* and *Suidae* families into “deer” and “boar” classes, respectively, resulting in a dataset with 9612 annotations in total. In order to balance the dataset, the oversampling approach was implemented and 4234 additional boar images were additionally augmented during training, which resulted in a total of 13236 training dataset annotations (see Table 2). The combined datasets were as follows:

- Caltech Camera Traps (CCT) [31].
- 7523423 Camera Traps [20].
- North America Camera Trap Images (NACTI) [13].
- WCS Camera Traps.
- Island Conservation Camera Traps.
- Channel Islands Camera Traps [32].
- ENA24-detection [33].
- Wellington Camera Traps [34].

### 4.3. Pre-Processing

**Figure 4:**
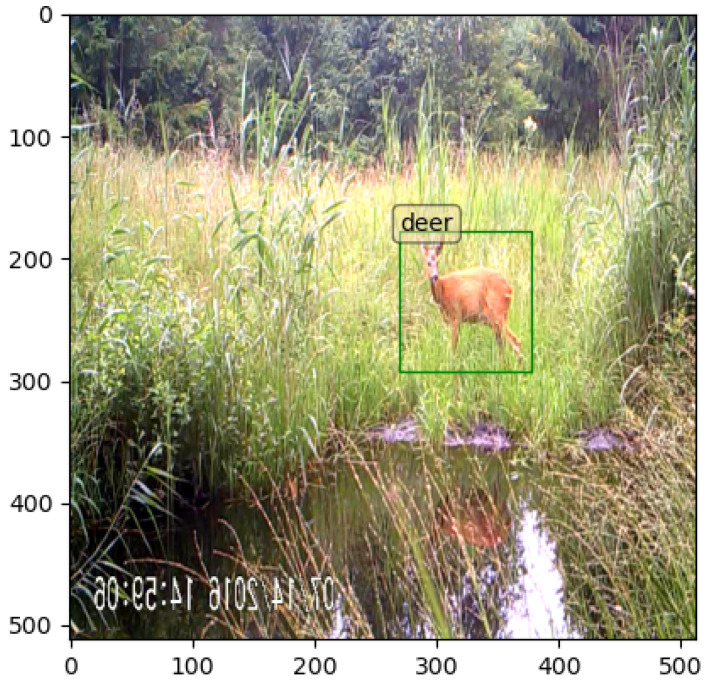
Example of the processed camera trap image with the visualized ground-truth bounding box.

The images were initially re-scaled to a 512 × 512 pixel size to lower the computational complexity of the convolution operations without losing much information of the object (comparing with the original size, it reduced convolution FLOPS ∼11 times [35]). On average, the animals of interest occupied ∼6.23% of the image, which was enough to conserve the textures when down-scaled ∼3.52 times. Several initial image resolutions were tested, but 512 × 512 was selected as optimal in terms of speed and accuracy and assumed to be good enough to capture an animal’s distinctive features (see Figure 4). After that, the images were normalized by subtracting the mean values from every pixel in the R, G and B channels and dividing it with standard deviation values to contribute to the convergence properties of the network [36].

Lastly, data augmentation was applied as mentioned in Section 4.2. Each image was flipped horizontally with 0.5 probability and random brightness, contrast and saturation variations in the non-extreme range were applied. These techniques simulate naturally occurring lighting variations, object pose transformations and thus contribute model robustness.

## 5. Experimental Results and Discussion

### 5.1. Experiments

In order to assess the quantitative effect of different learning approaches and compare different models, two experiments were designed. In all our experiments, we used the data that are described in Table 1 and Table 2 as our training and test data, respectively. The test data were not involved in the training and only used for evaluating performance. We selected the hyperparameters after our tests from the best performing values as follows:

1. Faster RCNN–ResNet50 network and RetinaNet were trained for 34,850 iterations (10 epochs) on the training dataset with a batch size of 4, learning rate of 0.0001 and Adam optimizer for the weight update. Other batch sizes and optimizers were tested, but those mentioned above were selected because they produced seemingly good convergence for the first training epoch.
2. To assess the effectiveness of the learning strategies, the RetinaNet results from the first experiment were compared with the control cases featuring RetinaNet without pre-trained weights and RetinaNet results for the corresponding number of iterations on the training set without augmentation.

### 5.2. Experiment 1

Faster R-CNN and RetinaNet were trained for ten epochs (34,850 iterations; 3485 iterations per epoch) on the training dataset; we updated their weights with each iteration. The results are represented in Table 3. The mAP (mean average precision) metric was used for model evaluation. The mAP for the 0.5 and 0.75 IoU threshold values and the averaged mAP for the IoU threshold values between 0.5 and 0.95 with a step size of 0.05 were measured. The best mAP@0.5:0.05:0.95 of 0.2786 was obtained by the Faster R-CNN–ResNet50 model already on the second epoch. However, after that, the Faster R-CNN’s mAP drastically decreased (almost by 0.1) during the three subsequent epochs and then slightly recovered, reaching 0.2582 in the 10th epoch. The best mAP@0.5:0.05:0.95 score for RetinaNet was 0.2659 which was obtained on the 7th epoch and is comparably close to the Faster-RCNN’s best result. Unlike Faster R-CNN, RetinaNet demonstrated more stable precision dynamics. After eight epochs, neither models’ precision improved explicitly.

**Figure 5:**
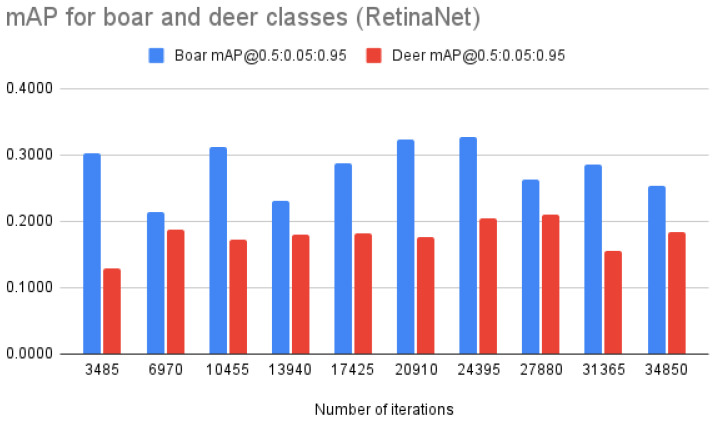
RetinaNet mAP@0.5:0.05:0.95 for “boar” and “deer” classes.

**Figure 6:**
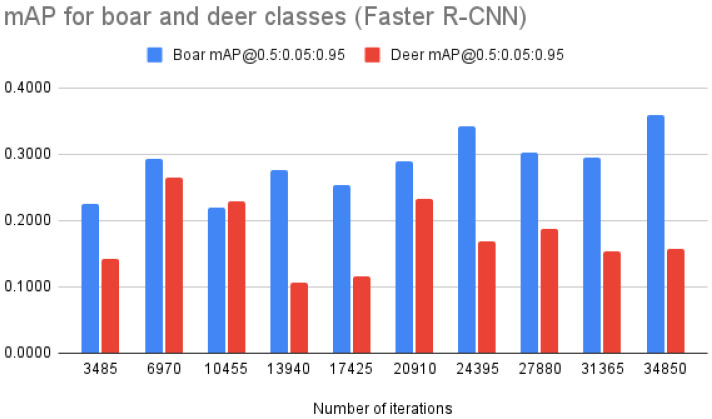
Faster R-CNN mAP@0.5:0.05:0.95 for “boar” and “deer” classes.

Per-class mAP dynamics for RetinaNet (see Figure 5) showed that the model was able to learn to detect boars more successfully, which was the main initial concern. A similar pattern was observed for Faster R-CNN (see Figure 6) with the detection of boars having higher precision (by ∼0.1 on average).

### 5.3. Experiment 2

To assure that the learning optimization techniques were effective, two additional control cases were tested, i.e., RetinaNet without pre-trained weights and pre-trained RetinaNet with non-over-sampled data (see Table 4).

Without pre-trained weights, RetinaNet performed the worst when trained for one epoch. The loss values for non-pre-trained network converged more slowly. In addition, the mAP@0.5 score was lower by 0.0751 and mAP@0.5:0.05:0.95 was lower by 0.0463. We can conclude that pre-trained network weights successfully increased the learning efficiency of the network.

RetinaNet trained on the non-oversampled dataset achieved larger per-class mAP score differences than pre-trained RetinaNet trained on the oversampled dataset, which contradicts with the intention of the oversampling implementation in this work. For the mAP@0.5:0.05:0.95 (see Table 4), the difference was 0.1742, compared with 0.0673 (∼2.5 times larger). This can be a temporal effect which would gradually dissolve, though it could also be the case whereby oversampling deteriorates the model precision. From Table 4, we can see that oversampling improved "boar" class accuracy, but drastically decreased "deer" class accuracy.

**Figure 7:**
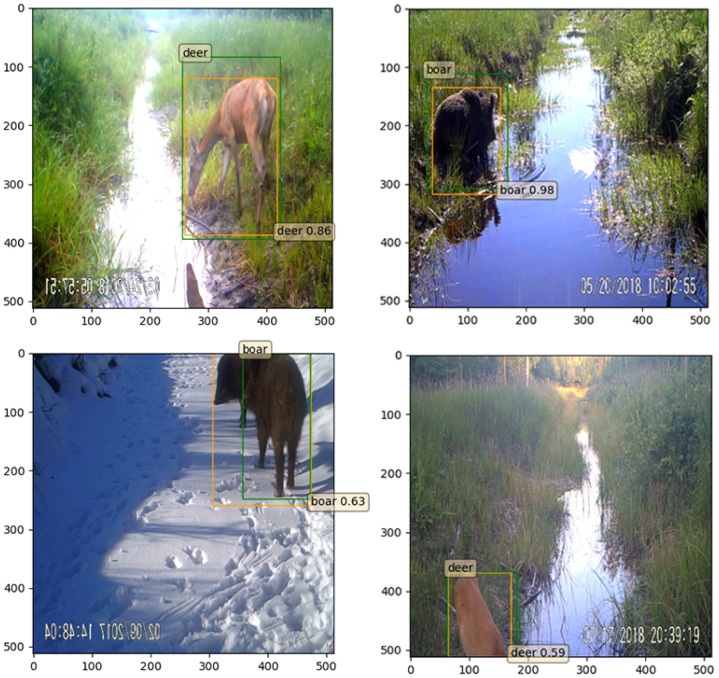
Prediction examples.

Faster R-CNN trained on the non-over-sampled data had uneven mAP scores for “deer” and “boar” compared to the first experiment’s results. Faster-RCNN from the first experiment showed almost even mAP for the “deer” and “boar” classes, which suggests that the model was able to learn in an unbiased manner and rare case oversampling was successful, as can be seen in Figure 7.

### 5.4. Discussion

The mAP evaluation suggested that both models successfully learned to detect “boar” and “deer" with average precision exceeding 25%. After the eighth epoch, the performance of both models started to decrease, which could be interpreted as overfitting (see Table 3). mAP differences for the “deer" and “moose" classes can be related to certain biases in the training and test sets and to the relatively small test-set size, which means that individual detection complications have a more significant impact on the precision result.

Various deer species presented in the dataset could visually look very different, such as roe deer, moose and red deer. This results in a challenge for a network in terms of characterization of the class. Moreover, deer are more frequently captured on cameras facing large open areas. As a result, deer are frequently captured on big distances, making deer look smaller, which provides less information for successful localization and classification. In addition, deer are more frequently captured in or near ponds, so these captures usually have clear deer reflections, which is sometimes included in the bounding box, resulting in a smaller IoU between the grounding box and predicted box. From the other perspective, boars are more frequently captured in close shots, which focus on a smaller region, thus more frequently producing images where boars are cropped out, which also limits high-precision detection.

We compared the results of our method with the state of the art in Table 5. We chose YOLOv4 and SSD for the comparison, since they are very popular state-of-the-art detection methods. The results show that our three-module method performed better than the others. Our method with RetinaNet as backbone performed the best for mAP@0.75, while Faster R-CNN as backbone performed the best for the remaining metrics. Our results are promising as we present them as a baseline for further research.

**Figure 8:**
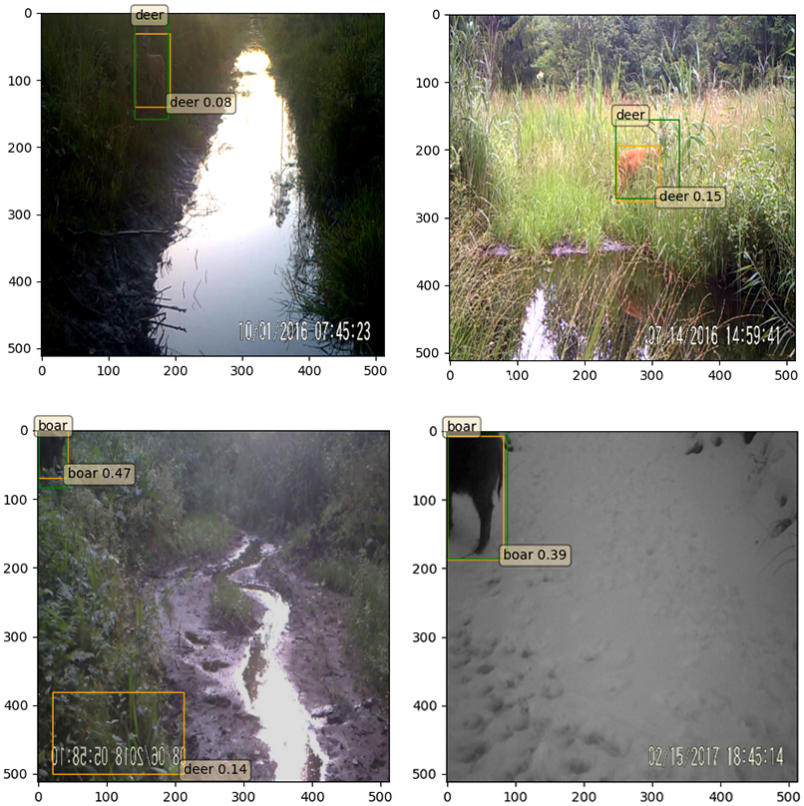
Samples of challenging captures.

The greatest limitation of our method is handling the outlier cases that come from image capturing in the wild by static camera systems. Since data were collected in the wild, there were situations where the captured image was not very clear for our method, for example, an animal behind the bushes in dark weather (See Figure 8). Another example is the images that contained animal reflections in water. We argue that the proposed method can be improved to overcome this limitation by including similar cases in the training data.

Our future steps include improving our dataset by adding more images for boar and deer species. Moreover, we are planning to annotate our collection of 8080 videos from the wildlife for the other species. In addition, we are aiming to improve our method to provide better generalization and performance.

## 6. Conclusions

Changes in the density of animal populations across an area have significant effects on both the wildlife and the human society in that environment. Overabundant wild ungulate populations cause damage to young forest stands and crop fields, as well as resulting in more frequent collisions with cars on roads, and have been reported as an increasing problem across the Europe [1]. A steady increase in wild ungulate populations has also been observed in Latvia over the last five years according to the State Forest Service data, e.g., +10% for elk, +22% for red deer and +44% for roe deer. High-frequency monitoring of the wild boar population is of interest due to the African Swine Fewer [37]. In both cases, camera trap networks could provide continuous monitoring of the spots of interest regardless of daytime and weather conditions. The effective management of the large data number acquired and its analysis are the main challenges in the wider uptake of camera traps in wild animal population monitoring. Automated data processing and animal species recognition is of high interest to the improvement of monitoring approaches. In this paper, we present a custom dataset that includes images of wild boar and deer that were collected in Latvia. Furthermore, we implemented two methods for the localization and classification of animals, using RetinaNet and Faster R-CNN as backbones. We discussed the optimization of the training and examined the impact of data augmentation. In conclusion, we created a baseline for the real world data collected in Latvia.

## Figures and Tables

**Table 1:** Test dataset demography.

Species or Group Name	Scientific Name	Number of Annotations
Deer	Cervidae	516
Wild boar	Sus Scrofa	526
Other species		86
Total count		1128

**Table 2:** Training dataset demography.

Species or Group Name	Scientific Name	Number of Annotations	Number of Augmented Samples	Total Number of Annotations
Deer	Cervidae	6970	0	6970
Wild boar	Sus Scrofa	2642	4328	6970
Total count		9612	4328	13,940

**Table 3:** Experiment 1: mAP evaluation.

Model	Metrics	Number of Iterations									
		3485	6970	10,455	13,940	17,425	20,910	24,395	27,880	31,365	34,850
Faster R-CNN	mAP @0.5:0.05:0.95	0.1832	**0.2697**	**0.2238**	**0.1913**	**0.1848**	**0.2618**	**0.2551**	**0.2449**	**0.2241**	**0.2582**
	mAP “deer”	0.1420	0.2584	0.2288	0.1062	0.1164	0.2336	0.1684	0.1877	0.1539	0.1576
	mAP “boar”	0.2244	0.2810	0.2187	0.2764	0.2532	0.2900	0.3417	0.3021	0.2942	0.3589
	mAP@0.5	0.3229	0.4561	0.3934	0.3305	0.3148	0.4562	0.4073	0.4065	0.3776	0.4204
	**mAP “deer”**	**0.2800**	**0.4737**	**0.4337**	**0.2154**	**0.2098**	**0.4332**	**0.3065**	**0.3414**	**0.2956**	**0.2996**
	mAP “boar”	**0.3657**	**0.4385**	**0.3531**	**0.4456**	**0.4197**	**0.4791**	**0.5080**	**0.4715**	**0.4596**	**0.5411**
	mAP @0.75	0.1932	0.2860	0.2229	0.1926	0.1959	0.2855	0.2758	0.2571	0.2488	0.2756
	**mAP “deer”**	**0.1367**	**0.2671**	**0.2222**	**0.0970**	**0.1175**	**0.2218**	**0.1659**	**0.1881**	**0.1454**	**0.1536**
	**mAP “boar”**	0.2496	0.3048	0.2235	0.2881	0.2743	0.3492	0.3857	0.3260	0.3521	0.3976
**RetinaNet**	**mAP @0.5:0.05:0.95**	**0.2158**	**0.2016**	**0.2413**	**0.2046**	**0.2346**	**0.2494**	**0.2659**	**0.2364**	**0.2202**	**0.2192**
	mAP “deer”	0.1287	0.1884	0.1715	0.1791	0.1827	0.1757	0.2053	0.2098	0.1551	0.1844
	mAP “boar”	0.3029	0.2148	0.3111	0.2301	0.2865	0.3231	0.3266	0.2630	0.2853	0.2540
	mAP@0.5	0.3740	0.3725	0.4133	0.3574	0.4134	0.4198	0.4364	0.4173	0.3738	0.3922
	**mAP “deer”**	**0.2727**	**0.3776**	**0.3361**	**0.3473**	**0.3530**	**0.3437**	**0.3814**	**0.4021**	**0.3017**	**0.3789**
	mAP “boar”	**0.4752**	**0.3673**	**0.4904**	**0.3675**	**0.4737**	**0.4959**	**0.4913**	**0.4325**	**0.4458**	**0.4054**
	mAP @0.75	0.1996	0.1909	0.2483	0.2179	0.2666	0.2678	0.2890	0.2421	0.2341	0.2236
	**mAP “deer”**	**0.1028**	**0.1473**	**0.1499**	**0.1642**	**0.1844**	**0.1631**	**0.2152**	**0.1929**	**0.1442**	**0.1556**
	mAP “boar”	0.2963	0.2345	0.3467	0.2716	0.3487	0.3724	0.3628	0.2913	0.3240	0.2915

**Table 4:** Experiment 2: mAP evaluation.

Metrics	Model		
	RetinaNet Pre-Trained	RetinaNet Not Pre-Trained	RetinaNet Pre-Trained (Non-Oversampled Dataset)
mAP @0.5:0.05:0.95	0.2158	0.1695	0.2290
mAP “deer”	0.1287	0.1492	0.1953
mAP “boar”	0.3029	0.1897	0.2626
mAP@0.5	0.3740	0.2989	0.4029
mAP “deer”	0.2727	0.2900	0.3758
mAP “boar”	0.4752	0.3078	0.4299
mAP @0.75	0.1996	0.1688	0.2265
mAP “deer”	0.1028	0.1441	0.1714
mAP “boar”	0.2963	0.1935	0.2815

**Table 5:** Comparison with the state of the art.

Metrics	Model			
	Ours with RetinaNet	Ours with Faster R-CNN	YOLOv4 [27]	SSD [28]
mAP @0.5:0.05:0.95	0.2659	0.2697	0.2295	0.2084
mAP @0.5	0.4364	0.4562	0.4010	0.3897
mAP @0.75	0.2890	0.2860	0.2545	0.2410

## Data Availability

Information about obtaining the dataset can be requested by contacting D. Jakovels at dainis.jakovels@vri.lv.
